# Facile, high efficiency immobilization of lipase enzyme on magnetic iron oxide nanoparticles via a biomimetic coating

**DOI:** 10.1186/1472-6750-11-63

**Published:** 2011-06-08

**Authors:** Yuhong Ren, Jose G Rivera, Lihong He, Harsha Kulkarni, Dong-Keun Lee, Phillip B Messersmith

**Affiliations:** 1 State Key Laboratory of Bioreactor Engineering, East China University of Science and Technology, 130 Meilong Road, Shanghai 200237, China; 2 Biomedical Engineering Department, Northwestern University, 2145 Sheridan Road, Evanston 60208, USA; 3 Chemistry of Life Processes Institute, Northwestern University, 2145 Sheridan Road, Evanston 60208, USA; 4 Chemical and Biological Engineering Department, Northwestern University, 2145 Sheridan Road, Evanston 60208, USA; 5 Materials Science and Engineering Department, Northwestern University, 2145 Sheridan Road, Evanston 60208, USA; 6 Institute for Bionanotechnology in Medicine, Northwestern University, 2145 Sheridan Road, Evanston 60208, USA; 7 Robert H. Lurie Comprehensive Cancer Center, Northwestern University, 2145 Sheridan Road, Evanston 60208, USA

## Abstract

**Background:**

Immobilization of lipase on appropriate solid supports is one way to improve their stability and activity, and can be reused for large scale applications. A sample, cost- effective and high loading capacity method is still challenging.

**Results:**

A facile method of lipase immobilization was developed in this study, by the use of polydopamine coated magnetic nanoparticles (PD-MNPs). Under optimal conditions, 73.9% of the available lipase was immobilized on PD-MNPs, yielding a lipase loading capacity as high as 429 mg/g. Enzyme assays revealed that lipase immobilized on PD-MNPs displayed enhanced pH and thermal stability compared to free lipase. Furthermore, lipase immobilized on PD-MNPs was easily isolated from the reaction medium by magnetic separation and retained more than 70% of initial activity after 21 repeated cycles of enzyme reaction followed by magnetic separation.

**Conclusions:**

Immobilization of enzyme onto magnetic iron oxide nanoparticles via poly-dopamine film is economical, facile and efficient.

## Background

Lipases (glycerol ester hydrolases E.C.3.1.1.3) are an important group of enzymes which have been widely used in the catalysis of different reactions [1,2]. These enzymes have been applied in chemical and pharmaceutical industrial applications due to their catalytic activity in both hydrolytic and synthetic reactions. However, free lipases are easily inactivated and difficult to recover for reuse. Therefore, especially in large-scale applications, lipases are often immobilized on solid supports in order to facilitate recovery and improve operational stability under a wide variety of reaction conditions. Some lipase immobilization strategies involve the conjugation of lipases via covalent attachment, cross-linking, adsorption and entrapment onto hydrophobic or hydrophilic polymeric and inorganic matrixes [3-5].

In recent years, magnetic nanoparticles (MNPs) based on iron oxides, have attracted much interest thanks to their multifunctional properties, such as biocompatibility, superparamagnetism, small size and low toxicity [6]. They have been applied in magnetic resonance imaging (MRI) [7], biosensors [8] and as anti-cancer drugs carriers [9]. Due to their high specific surface area and easy separation from the reaction medium by the use of a magnet, they have been employed in enzymatic catalysis applications [10,11]. Typical strategies for immobilizing lipase onto MNPs rely on surface grafting via low molecular weight linkers or polymers containing amino or epoxy functional groups to which lipases are reacted via covalent conjugation methods [1,11]. Using such methods, the maximum reported loading capacity of lipase on nanoparticles is approximately 130 mg/g, using a complex methodology [1]. One drawback of existing lipase immobilization technologies is that the activity of lipases decreases significantly upon immobilization due possibly to changes in enzyme secondary structure, or limited access of substrate to the active site of the surface bound enzyme [12]. Thus, despite numerous reported approaches for immobilization of lipases on magnetic nanoparticles, there is still the need for simple, cost-effective and high loading capacity methods.

In this work, we present a facile, biomimetic approach to immobilize lipases onto iron oxide MNP surfaces modified with polydopamine, an in-situ formed coating inspired by the adhesive proteins secreted by marine mussels [13]. The ortho-dihydroxyphenyl (catechol) functional group found in dopamine is also present in mussel adhesive proteins in the form of the amino acid DOPA, where it is highly adhesive to oxide surfaces [14,15] and under alkaline conditions oxidizes to form quinone, a species that is reactive toward nucleophiles such as primary amines. Dopamine, containing both catechol and primary amine, was previously found to produce conformal coatings on surfaces by self-polymerization [13], which are further capable of immobilizing biomolecules [15]. In the method described here, polydopamine serves as a conformal coating for the purposes of lipase immobilization onto MNPs. Our results demonstrate that PD-MNPs exhibit high efficiency for lipase immobilization under aqueous conditions, and the enzyme retains high activity after many cycles of magnetic separation and reuse.

## Results and discussion

### Overall Strategy for Preparing Lipase Immobilized Iron Oxide MNPs

**Figure 1:**
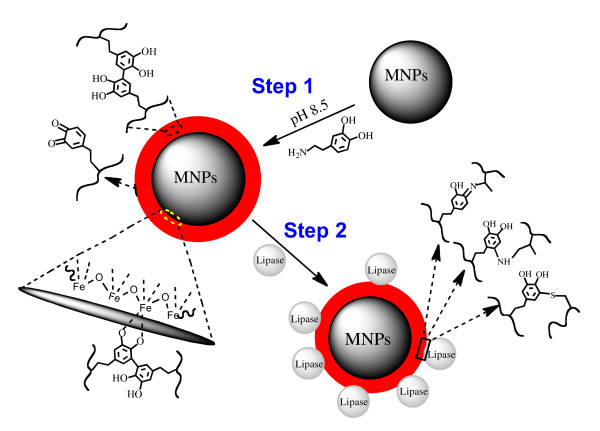
**Two-step synthesis of lipase immobilized PD-MNPs**. Two-step synthesis of lipase immobilized PD-MNPs.

Our two-step method of polydopamine surface modification and lipase immobilization is illustrated in Figure 1. Iron oxide MNPs prepared by alkaline co-precipitation of Fe(II) and Fe(III) were first incubated in an alkaline dopamine solution for several hours to create an adherent polydopamine film on MNPs (PD-MNPs), after which enzyme was immobilized by exposure of PD-MNPs to a lipase containing solution.

### Synthesis and characterization of MNPs and PD-MNPs

Catechol derivatized polymers have been previously employed for grafting functional polymers onto surfaces of MNPs [16-18]. In the present work we took advantage of dopamine self-polymerization to form MNPs with polydopamine coatings. In-situ polymerization of dopamine represents a versatile method for modifying solid surfaces with adherent coatings for a variety of functional purposes [13]. The reactions that occur in an alkaline dopamine solution are not unlike those that occur during melanin formation, beginning with oxidation of catechol to yield dopamine quinone, which in turn participates in a series of further intra- and intermolecular reactions that ultimately give rise to a high molecular weight heterogeneous polymer (polydopamine). Polydopamine formation occurs both in solution and as a conformal coating on surfaces. In the present case involving iron oxide MNPs, the polydopamine is likely bound to the surface and even nucleated by dopamine molecules that strongly interact with the oxide surface [14].

**Figure 2:**
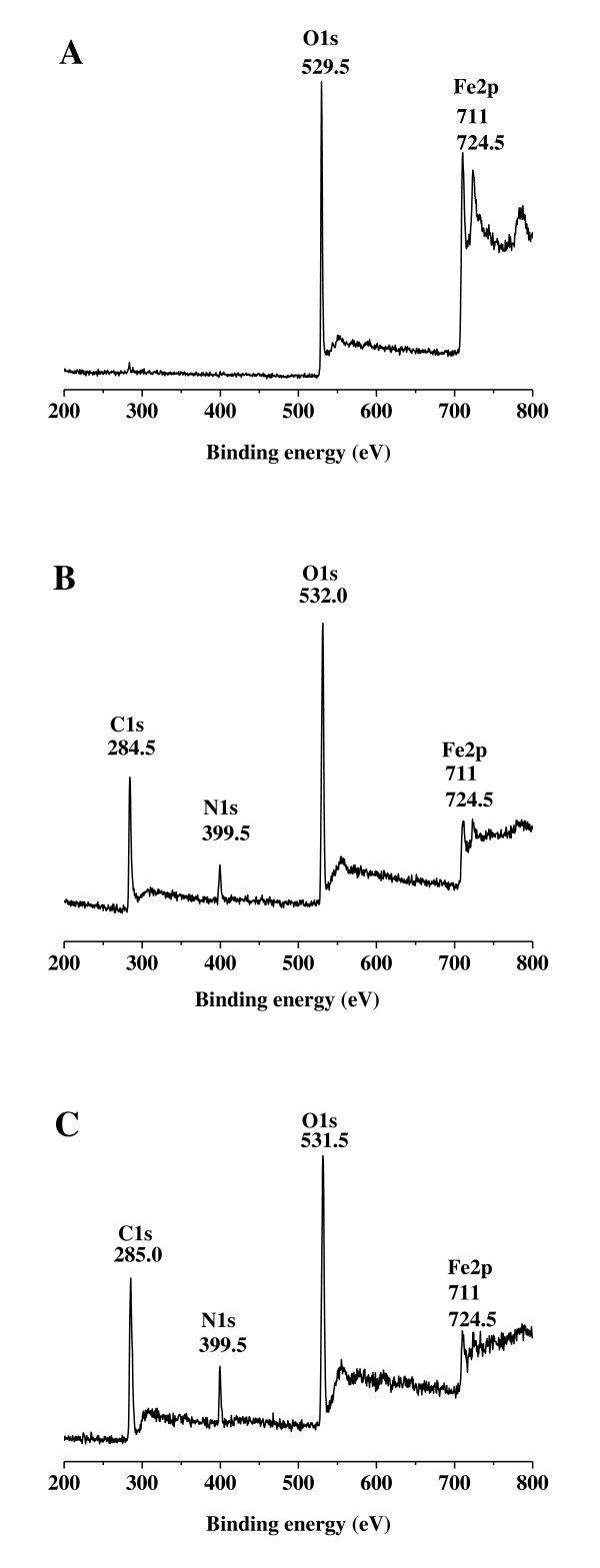
**XPS spectra**. XPS spectra of MNPs (A), PD-MNPs (B), and lipase immobilized PD-MNPs (C).

X-ray photoelectron spectroscopy (XPS) spectra of the MNPs and PD-MNPs are shown in Figure 2. Whereas the unmodified MNPs contained little carbon and nitrogen content (Figure 2A), the XPS spectrum of PD-MNPs contained prominent peaks correlating to C1s (284.5 eV) and N1s (399.5 eV) (Figure 2B). The calculated nitrogen-to-carbon (N/C) ratio of the PD-MNPs is 0.119, which is close to the theoretical N/C of 0.125 for dopamine. Additionally, the Fe 2p (711 and 724.5 eV) peaks are attenuated in PD-MNPs compared to MNPs due to the presence of the polydopamine coating. Further evidence for polydopamine coating is provided by a shift of the O1s peak from 529.5 eV in MNPs and corresponding to iron oxide, to 532 eV corresponding to organic oxygen.

**Figure 3:**
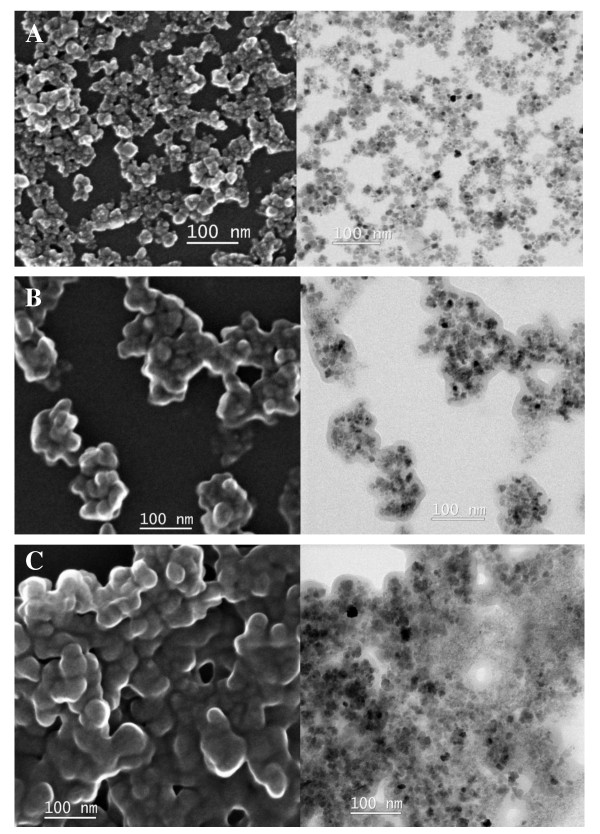
**SEM and TEM spectra**. SEM (left column) and TEM (right column) images of MNPs (A), PD-MNPs (B), and lipase immobilized PD-MNPs (C). The PD-MNPs were prepared using 2.5 mg/ml initial dopamine and lipase immobilization performed using 5 mg/ml initial lipase and 10 mg/ml PD-MNPs concentration.

The size and morphology of the MNPs and the PD-MNPs were observed by SEM and TEM (Figure 3A and 3B). The unmodified MNPs were composed of clusters of nanoparticles with sizes ranging from 5 nm to 15 nm. In comparison, SEM and TEM images of the PD-MNPs reveal aggregates iron oxide particles entrapped in a conformal polydopamine coating of approximate thickness 10 nm. The average size of the PD-MNPs agglomerates ranged from 50 nm to 250 nm. Although some observed aggregation could be caused by drying during TEM sample preparation [19], it is also possible that polydopamine induced partial aggregation of MNP clusters.

**Figure 4:**
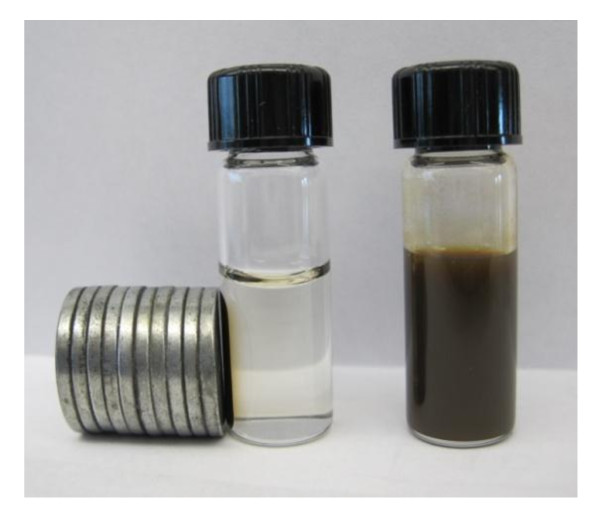
**Magnetic isolation of PD-MNP**. Photographs of an aqueous suspension of PD-MNPs before (right) and after (left) magnetic isolation.

Magnetic isolation of PD-MNPs was accomplished by placing a magnet adjacent to a vial containing PD-MNPs dispersed in an aqueous medium (Figure 4). Within two minutes the solution became clear as a result of movement of the PD-MNPs towards the magnet, demonstrating simple and rapid isolation of PD-MNPs using a magnetic field. Removal of the magnet followed by agitation led to re-suspension of the PD-MNPs.

### Lipase immobilization onto PD-MNPs

After formation, the polydopamine coating contains residual quinones that are reactive toward nucleophilic groups, affording covalent immobilization of polymers and biomolecules through Michael addition and/or Schiff base formation [13,15]. In the present case, similar reactions between the active quinone groups at the surface of the PD-MNPs and the amino or thiol groups of the lipase enzyme result in lipase immobilization onto the surfaces of the PD-MNPs during the lipase immobilization step (Figure 1). The reaction between PD-MNPs and lipase was spontaneous and rapid, resulting in precipitation of lipase-containing MNPs particles soon after mixing. Analysis of free protein concentration in solution during lipase immobilization at 4°C showed little change after 2 hours, further confirming the completion of the immobilization reaction.

XPS analysis of the lipase immobilized PD-MNPs (Figure 2C) showed increases in the N1s peak (399.5 eV) and the calculated N/C ratio (0.139) compared to PD-MNPs, reflecting the higher nitrogen content of lipase compared to polydopamine. Little change in aggregate appearance and morphology was observed upon immobilization of lipase onto PD-MNPs, although SEM and TEM analysis (Figure 3C) revealed further agglomeration (average size 150 - 250 nm). It is not known whether the additional aggregation occurred during lipase immobilization or was an artifact of EM sample preparation.

Lipase immobilization efficiency as well as the activity of bound enzyme was found to be dependent on conditions used during preparation of PD-MNPs and immobilization of lipase (Table 1). In the absence of polydopamine treatment, binding of lipase to MNPs was inefficient and resulted in low activity. Through the use of polydopamine, the amount of bound lipase was increased by as much as 3-fold. Increasing the concentration of dopamine used in preparing the PD-MNPs in the range 0.25 - 2.5 mg/ml resulted in an increase in bound lipase from 6.96 mg to 9.16 mg coupled with an increase in enzyme specific activity from 2.62 to 6.44 U/mg. These results show that polydopamine is essential for immobilization of high amounts of lipase enzyme onto MNPs. However, taking into consideration the cost of enzyme and that specific activity of bound lipase did not significantly increase above initial dopamine concentration of 2.5 mg/ml, we identified optimum conditions to be polydopamine deposition at 2.5 mg/ml dopamine concentration followed by lipase immobilization at a 2:1 (w/w) ratio of PD-MNPs to lipase. Under these conditions we achieved a lipase loading of 429 mg/g of material (85.8% of the amount of added lipase) with 8.78 U/mg specific activity (73.9% of free lipase specific activity), much higher than previous reports [1].

**Table 1:** Effect of preparation conditions on lipase immobilization efficiency and activity of lipase immobilized PD-MNP

Dopamine (mg/ml)	PD-MNP (mg)	Lipase (mg)	Bound Lipase (mg)	Activity (U)	Specific Activity (U/mg lipase)
0	10	10	3.16 ± 0.52	9.75 ± 2.28	3.09 ± 0.16
0.25	10	10	6.96 ± 0.49	18.19 ± 2.90	2.62 ± 0.13
0.5	10	10	7.81 ± 0.62	21.08 ± 2.49	2.70 ± 0.17
1.0	10	10	8.26 ± 0.48	28.81 ± 3.08	3.49 ± 0.17
2.5	10	10	9.17 ± 0.58	59.00 ± 3.39	6.44 ± 0.38
3.75	10	10	9.37 ± 0.55	62.50 ± 3.04	6.67 ± 0.37
5.0	10	10	9.46 ± 0.57	64.89 ± 2.69	6.86 ± 0.40
2.5	10	5	4.29 ± 0.22	37.65 ± 2.52	8.78 ± 0.33
Free lipase		10		118.76 ± 4.13	11.88 ± 0.41

Effect of preparation conditions on lipase immobilization efficiency and activity of lipase immobilized PD-MNP (n = 3).

### Effect of temperature and pH on free and immobilized lipase activity

**Figure 5:**
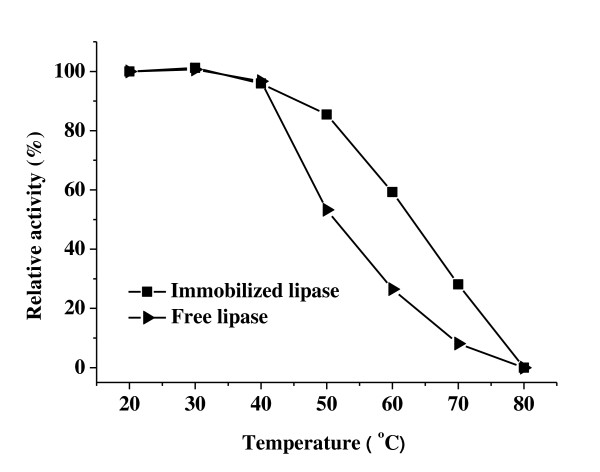
**The effect of temperature on the activity of free and immobilized lipase**. Activity was measured at 37°C after treatment for 1 hour in phosphate buffer (10 mM, pH 7.0) at the temperatures indicated. The PD-MNPs were prepared using 2.5 mg/ml initial dopamine and lipase immobilization performed using 5 mg/ml initial lipase and 10 mg/ml PD-MNPs concentration.

The effect of temperature on the activity of free and immobilized lipase at pH 7.0 is shown in Figure 5. The activity of both free and immobilized lipase is not adversely affected at temperatures below 40°C. The relative activity of free lipase dropped significantly above 40°C and decreased to just 26.5% of the initial activity at 60°C. In comparison, lipase immobilized on PD-MNPs retained 59.3% of activity at 60°C. A possible explanation for this is enhanced thermal stability in the immobilized state, leading to less denaturation of protein [10].

**Figure 6:**
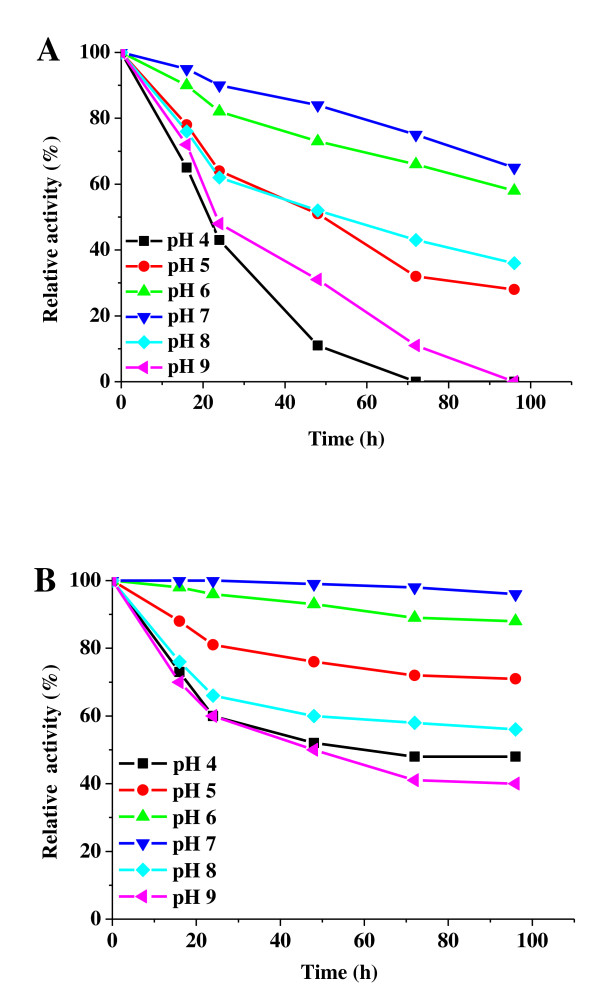
**The effect of pH on the activity of free and immobilized lipase**. The effect of pH on the activity of free (A) and immobilized (B) lipase. The samples were pre-incubated in phosphate buffer (10 mM) at the pH values and times indicated before determining enzyme activity at pH 7.0 and 37°C. The PD-MNPs were prepared using 2.5 mg/ml initial dopamine and lipase immobilization performed using 5 mg/ml initial lipase and 10 mg/ml PD-MNPs concentration.

The effect of pH on the relative activity of free and immobilized lipase is shown in Figure 6. According to our data, the activity of lipase immobilized on PD-MNPs was retained throughout a wider pH range compared to free enzyme. At pH 6-7, free lipase was stable for short incubation periods but was adversely affected at longer incubation times, retaining only 60-70% of the activity after 96 h incubation. At pH 5 and 8 only 30-40% of the activity of the free enzyme was retained after 96 h, and complete loss in activity was observed for free lipase upon incubation at pH 4 and 9. In contrast, PD- lipase immobilized on MNPs retained more than 90% of activity at pH 6 and 7, and even retained more than 50% of activity after 96 hours incubation at pH 4, 5 and 8. The increased stability of immobilized lipase may result from multipoint covalent linking between lipase and the PD-MNPs, which prevents lipase denaturation in acid or alkaline environments [10,20].

### Magnetic Isolation and Reuse

**Figure 7:**
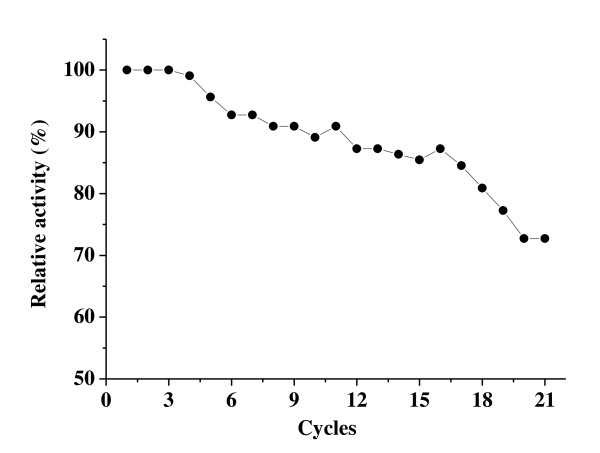
**The activity of immobilized lipase after multiple cycles of magnetic isolation and reuse**. Activity of lipase immobilized PD-MNPs after multiple cycles of magnetic isolation and reuse. The PD-MNPs were prepared using 2.5 mg/ml initial dopamine and lipase immobilization performed using 5 mg/ml initial lipase and 10 mg/ml PD-MNPs concentration. The total elapsed time of the experiment was less than 10 hours.

Reuse of enzymes in industrial processes is advantageous from an economic point of view. We therefore investigated this feature in lipase immobilized PD-MNPs, whose magnetic properties and enhanced enzyme stability were designed to facilitate multiple cycles of magnetic isolation and reuse. Figure 7 shows the activity of lipase immobilized PD-MNPs after multiple cycles of magnetic separation and reuse. Although the activity of the immobilized lipase began to decrease after 4 cycles, more than 70% of its initial activity was still retained after 21 cycles. The decrease of activity may be caused by aggregation and loss of particles during magnetic isolation, denaturation of protein and the gradual loss of lipase from the PD-MNPs.

## Conclusions

In this work, we described a facile method to immobilize lipase onto magnetic nanoparticles through an adhesive polydopamine film. Our immobilization experiments show that the PD-MNPs exhibit high lipase loading capacity due to high surface area and strong adhesive interactions between lipase and polydopamine. In addition, the immobilized lipase shows high specific activity and favorable thermal and pH stability compared to free lipase. Importantly, the lipase immobilized PD-MNPs display good reusability as well as the convenience to be magnetically recovered. These results confirm that immobilization of enzyme onto magnetic iron oxide nanoparticles by poly-dopamine film, is economical, facile and efficient.

## Methods

### Materials

*Candida rugosa *lipase type VII (E.C.3.1.1.3), ferric chloride hexahydrate (FeCl_3_·6H_2_O), ferrous chloride tetrahydrate (FeCl_2_·4H_2_O), dopamine hydrochloride, tributyrin, gum acacia, tris buffer and sodium hydroxide were purchased from Sigma-Aldrich (USA). Ultrapure water (resistivity = 18.2 MΩ, pH 6.82) was used in all experiments and obtained from a NANOpure Infinity^@ ^system from Barnstead/Thermolyne Corporation (Dubuque, IA).

### Synthesis and surface modification of MNPs

Figure 1 presents the synthesis and modification of MNPs. MNPs were prepared by the conventional co-precipitation method [21] with some modifications. Briefly, 0.5 mmol FeCl_2 _and 1.0 mmol FeCl_3 _were dissolved in 50 ml ultrapure water under nitrogen at room temperature, then the pH of the solution was adjusted to 10.0 using 6.0 M NaOH under vigorous stirring. After stirring for 2 hours, the magnetite precipitates were separated and washed several times with ultrapure water by magnetic decantation. The precipitate was dispersed in 50 ml Tris buffer (10 mM, pH 8.5) under ultrasonication for 15 min, after which large precipitates were removed. Dopamine hydrochloride (125 mg, 2.5 mg/ml) was added to the remaining MNPs suspension with vigorous stirring, and the pH of the solution was kept at 8.5 by addition of 10 mM NaOH. After 3 hours, the PD-MNPs was collected by magnetic decantation and washed 5 times with ultrapure water, and finally re-dispersed in ultrapure water to 2 mg PD-MNPs per milliliter solution by ultrasonication for 15 min.

### Immobilization of lipase

Lipase immobilization was carried out by adding the suspension of PD-MNPs in ultrapure water to a buffered enzyme solution. An aqueous solution of lipase (2 mg/ml) was prepared by dissolving the lyophilized enzyme in sodium phosphate buffer (10 mM, pH 7.0) solution. A fresh solution of the previously described PD-MNPs (5 ml, 2 mg/ml) was added to the lipase solution (5 ml, 2 mg/ml) at 4°C. Precipitation was observed immediately as the two solutions mixed. After shaking at 180 rpm for 3 h (at 4°C), the lipase-loaded precipitates were collected and washed 3 times with ultrapure water and stored at 4°C prior to use. The protein concentration in the lipase solution before and after immobilization was determined using the Bradford method [22]. The difference in protein concentration was used to calculate the loading of lipase onto the PD-MNPs.

### Characterization of MNPs and PD-MNPs

XPS spectra were obtained using an Omicron ESCALAB (Omicron, Taunusstein, Germany) with a monochromatic Al Kα (1486.8 eV) 300-W X-ray source, a flood gun to counter charging effects, and ultrahigh vacuum (~10^-9 ^torr). The takeoff angle was fixed at 45°. Substrates were mounted on sample studs by means of double-sided adhesive tape. All binding energies were calibrated using the C1s peak (284.5 eV). Scanning electron microscopy (SEM) and transmission electron microscopy (TEM) images were acquired on a Hitachi HD2300 electron microscope (Hitachi, Japan) operated at 200 kV. TEM specimens were prepared by casting drops of dilute dispersion of nanoparticles aqueous solution on 200-mesh carbon coated copper grids (Ted Pella).

### Activity assay of free and immobilized lipase

The enzymatic activities of free and immobilized lipase were measured by titration of the organic acid that results from the hydrolysis of the tributyrin ester [23]. Briefly, the activity of the free lipase was assayed by adding 0.5 ml of free lipase (5 mg/ml, w/v) in phosphate buffer (10 mM, pH 7.0), 20 ml of a tributyrin solution consisting of 1% tributyrin as the substrate with 1% gum acacia as emulsifier. After 20 min of incubation at 37°C with shaking at 150 rpm, the reaction was stopped by the addition of 20 ml of ethanol. Immediately, the mixture was titrated against 50 mM NaOH in ethanol solution using phenolphthalein as indicator. The immobilized lipase activity was determined as described above by adding a known amount of lipase immobilized PD-MNPs. The lipase loaded PD-MNPs were sonicated for 10 min at 4°C before combining with substrate solution. After incubation of the mixture at 37°C for 20 min with shaking at 200 rpm, the lipase PD-MNPs were separated by a magnet, and the supernatant titrated using an NaOH in ethanol solution. One unit of lipase activity was defined as the amount of lipase which liberated 1 μmol of free acid per minute under the assay conditions. All activity measurements were carried out at least three times and the experimental error was less than 3%.

### Effect of temperature and pH

The effect of temperature on the free and immobilized enzyme activity was determined by pre-incubating in phosphate buffer (10 mM, pH 7.0) at temperatures ranging from 20°C to 80°C for 1 h, followed by measurement of the residual enzyme activity at 37°C as described above. The effect of pH on the activity of the free and immobilized enzyme was determined by pre-incubating at room temperature in phosphate buffer (10 mM) at pH ranging from 4 to 9, followed by determination of enzymatic activity at pH 7.0 as described in activity assay section. The residual activity of the immobilized lipase was normalized to the initial value determined at 37°C, pH 7.0 (the initial activity was defined as 100%).

### Recovery and reuse of immobilized lipase

The stability of immobilized lipase under conditions of repeated magnetic isolation and reuse was studied under the same conditions as described in activity assay section. After each enzyme run, the lipase containing PD-MNPs were magnetically isolated and washed twice with hexane and ultrapure water to remove any remaining substrate and product species before the next experiment. The residual activity of the immobilized lipase after each cycle was normalized to the initial value (the initial activity was defined as 100%).

## Authors' contributions

YR preformed most of experiments, participated in all data analysis, and drafted much of the manuscript. JGR, LH and DKL provided regular advice as the study progressed. HK preformed XPS experiments. PBM conceived of the study, participated in all data analysis, and drafted parts of the manuscript. All authors read and approved the final manuscript.
